# The new mechanism of cognitive decline induced by hypertension: High homocysteine-mediated aberrant DNA methylation

**DOI:** 10.3389/fcvm.2022.928701

**Published:** 2022-10-24

**Authors:** Chong Wan, Rui-Yi Zong, Xing-Shu Chen

**Affiliations:** ^1^Department of Military Medical Geography, Army Medical Training Base, Army Medical University (Third Military Medical University), Chongqing, China; ^2^College of Basic Medicine, Army Medical University, Chongqing, China; ^3^NCO School, Army Medical University, Shijiazhuang, China

**Keywords:** hypertension, cognition decline, DNA methylation, Hcy, Met

## Abstract

The prevalence and severity of hypertension-induced cognitive impairment increase with the prolonging of hypertension. The mechanisms of cognitive impairment induced by hypertension primarily include cerebral blood flow perfusion imbalance, white and gray matter injury with blood–brain barrier disruption, neuroinflammation and amyloid-beta deposition, genetic polymorphisms and variants, and instability of blood pressure. High homocysteine (HHcy) is an independent risk factor for hypertension that also increases the risk of developing early cognitive impairment. Homocysteine (Hcy) levels increase in patients with cognitive impairment induced by hypertension. This review summarizes a new mechanism whereby HHcy-mediated aberrant DNA methylation and exacerbate hypertension. It involves changes in Hcy-dependent DNA methylation products, such as methionine adenosyltransferase, DNA methyltransferases, *S*-adenosylmethionine, *S*-adenosylhomocysteine, and methylenetetrahydrofolate reductase (MTHFR). The mechanism also involves DNA methylation changes in the genes of hypertension patients, such as brain-derived neurotrophic factor, apolipoprotein E4, and estrogen receptor alpha, which contribute to learning, memory, and attention deficits. Studies have shown that methionine (Met) induces hypertension in mice. Moreover, DNA hypermethylation leads to cognitive behavioral changes alongside oligodendroglial and/or myelin deficits in Met-induced mice. Taken together, these studies demonstrate that DNA methylation regulates cognitive dysfunction in patients with hypertension. A better understanding of the function and mechanism underlying the effect of Hcy-dependent DNA methylation on hypertension-induced cognitive impairment will be valuable for early diagnosis, interventions, and prevention of further cognitive defects induced by hypertension.

## Introduction

The prevalence of hypertension is increasing rapidly worldwide. More than 1.5 billion individuals currently experience hypertension (1). Hypertension not only affects the cerebral vasculature, parenchyma, and metabolism but is also related to an increased risk of cognitive decline and vascular cognitive impairment (1–3). Moreover, hypertension has been shown to induce cognitive decline and accelerate mild cognitive impairment (MCI), progressive deterioration, and dementia (4–7).

Hypertension-induced cognitive decline includes attention, memory, and decision-making impairments across all age groups from young individuals, middle-aged adults, and older adults (1, 8–11). Young hypertensive individuals can experience cognitive capacity-related changes (8). Cross-sectional studies in older adults have shown that hypertension contributes to cognitive decline, including simple attention, executive function, and psychomotor speed (11, 12). In older adults, hypertension, as a marker of cognitive impairment, affects cognitive performance (13, 14). Furthermore, studies have shown a relationship between blood pressure (BP) and cognitive function (15). Having an optimal BP range is associated with the maintenance of cognitive function (16). White coat hypertension and borderline hypertension groups exhibited poorer cognitive ability than the normal BP group (17). In addition, antihypertensive medications, such as calcium channel blockers, induce a protective effect against cognitive impairment in patients with hypertension (11, 18, 19).

Neuroimaging results have demonstrated that hypertension results in cognitive decline and neurostructural changes (20). The brains of hypertensive patients have higher amounts of amyloid-beta (Aβ) plaques, atrophy, and neurofibrillary tangles than healthy controls. Moreover, positron emission tomography (PET) studies have shown that hypertension is a risk factor for Aβ deposits and glucose metabolism disorders. Furthermore, numerous studies have used diffusion tensor imaging to show that hypertensive patients with small vessel disease have white matter changes (20–23). Hypertension is also related to cerebral microbleeds as demonstrated using brain magnetic resonance imaging (MRI) (24). A fluorodeoxyglucose-PET imaging study in older adult hypertensive patients revealed that high white matter signal is associated with low metabolism in gray matter, which may explain the memory impairment exhibited by these patients (25).

The renin–angiotensin–aldosterone system (RAAS) is central to BP regulation and plays an important role in the central nervous system. Inhibition of the RAAS can reduce the rate of cognitive decline in patients with MCI and dementia (26, 27). Angiotensin-converting enzyme (ACE) influences the metabolism of angiotensin in the RAAS system, and ACE inhibitors suppress microglial activation and preserve dendritic integrity and cognitive function (28).

Angiotensin II-treated mice exhibit short-term memory impairment and greater blood–brain barrier (BBB) leakage, microglial activation, and myelin loss than control adult mice (29). In addition, gradual induction of angiotensin II-dependent hypertension has been shown to produce significant spatial learning impairments in middle-aged cytochrome P450 family 1, subfamily A polypeptide 1 (Cyp1a1)-Ren2 transgenic rats (30). Furthermore, increased plasma aldosterone impairs cognitive function, whereas spironolactone and eplerenone, mineral corticoid receptor blockers, protect against cardiovascular mortality and cognitive impairment (31, 32). The activity of cytochrome *c* oxidase in various brain regions in mice with portal hypertension has been confirmed experimentally and has demonstrated that high pressure affects brain metabolic activity and spatial memory in varying degrees (33). Studies have also shown that the time of stimulus input in hippocampal slices of hypertensive mice correlates with decreased mRNA expression of several genes, such as brain-derived neurotrophic factor (BDNF), Homer1, and disks large homolog 4 (34).

## Cognitive decline induced by hypertension

The supply of nutrients to the brain depends on dynamic blood flow. BP instability and abnormal blood flow and metabolism in patients with hypertension can damage neurons and impair cognitive function (35). There have been several advances in our understanding of the mechanisms underlying hypertension-induced cognitive decline, which include cerebral blood flow perfusion imbalance in response to cognitive and metabolic challenges, gray matter injury as reflected by changes in gray matter volume, cortical thinning, BBB dysfunction, Aβ accumulation, white matter injury, and genetic factors (7, 36). High homocysteine (HHcy) is one of the independent risk factors for hypertension that increases the risk of developing cognitive impairment (37).

### Cerebral blood flow perfusion imbalance

In hypertensive patients, the cerebral blood supply can become damaged due to the destruction of the structural and functional integrity of the cerebral microcirculation, which results in greater sparsity of the microvasculature, endothelial dysfunction, and neurovascular uncoupling (7, 38, 39). Studies have suggested that disturbances in cerebral perfusion are one mechanism by which hypertension impacts cognitive decline. In the temporal and occipital lobes of hypertensive patients, both cerebral perfusion and total and local cortical thicknesses are decreased (39, 40). Elevated BP (both acute and sustained) results in the extravasation of fluid and damage to cerebral blood vessels (38). Moreover, endothelial dysfunction and abnormal cerebral microcirculation cause cognitive impairment in patients with hypertension (41). Furthermore, hypertension is associated with elevated levels of circulating endothelial microvesicles that contribute to BP-related endothelial dysfunction, which may serve as a biomarker for hypertension-induced cognitive impairment (41, 42).

Disruptions in neurovascular coupling, which forms the basis for the relationships among neuronal activity, hemodynamic factors, and cell-to-cell signaling, may lead to a decrease in vascular reserve capacity and cognitive decline in patients with either hyper- or hypotension (43). Elevated BP in hypertensive patients causes vascular dysfunction, which affects cerebrovascular blood regulation (especially microvascular circulation) and leads to cognitive decline (41). Hypertension can accelerate cerebral blood flow, reduce metabolism, and decrease glucose utilization (44). Accordingly, the interplay between functional blood flow reorganization and vascular brain damage results in hypertension-related cognitive decline. Indeed, microvascular dysfunction has been shown to be responsible for deficits in memory and processing speed and cognitive decline (45).

### Gray matter injury

Gray matter disruptions are directly associated with cognitive impairment. Hypertension not only impairs the cardiovascular–renal axis but also impairs learning and memory (29). Spatial memory and cognition rely on the hippocampus, which is significantly smaller in hypertensive mice than in healthy mice (46). Hippocampal atrophy has been shown to be associated with hypertension; moreover, reduced dentate gyrus volume is associated with hypertension-induced cognitive impairment (47, 48). Hypertension impairs hippocampal neurogenesis in adult mice, affecting CA1 neurons, dendritic arborization, and long-term memory, which may be related to the down-regulation of the BDNF signaling pathway (49).

Hypertension damages the major arteries and capillaries in the brain, which, in turn, destroys the BBB and increases vascular permeability and accumulation of Aβ in the brain parenchyma (50, 51). Numerous studies have shown that MCI patients have accelerated destruction of the BBB and that blood vessel leakage accelerates hippocampal atrophy and neuronal loss, which leads to the onset of Alzheimer’s disease (AD) (52). A recent study showed that individuals with early cognitive impairment have brain capillary injuries. BBB destruction is an early biomarker of cognitive impairment that does not depend on Aβ or tau. Vascular contributions to cognitive impairment are increasingly being recognized (53, 54). Moreover, several angiodynamic changes can lead to cognitive decline. Studies in mice with cerebral venous congestion and BBB destruction have shown exacerbation of the neuroinflammatory response (51–54). Aβ promotes the overproduction of free radicals in endothelial cells, which results in neuronal cell necrosis. A study that explored the association between hypertension and AD using a hypertensive model of transverse aortic contraction found that the receptor for advanced cerebrovascular glycation end product activation is a key factor in the pathogenesis of AD. However, AD can be effectively suppressed by inhibiting this target, which offers potential as a new therapeutic option (50, 55). Another study showed that angiotensin II infusion causes learning and spatial memory deficits and anxiety, from the third week of perfusion, with Aβ deposition occurring in the fourth week (56).

### White matter injury

In white matter, oligodendrocytes wrap the axons of neurons to form the myelin sheath to maintain appropriate nerve impulse conduction, which plays an important role in cognitive function (57). Endothelial cells or the permeability of brain cells produces change, which results in cerebral vascular injury or structural changes in the arterioles of white matter. In hypertensive patients, decreased cerebral blood flow is often followed by ischemic infarction, white matter damage, and brain atrophy (58–60). White matter lesions (WMLs), including subcortical and/or periventricular white matter loosening, are present in 85% of hypertensive patients (61).

A significant positive correlation exists between BP and white matter abnormalities in patients with hypertension. BP at baseline is closely related to WML volume, which is also associated with hypertension in most brain regions (62). Furthermore, white matter damage destroys cortical and subcortical connections, which leads to the disconnection of various functional areas, affecting the cognitive function of hypertensive patients (63). In a long-term study on white matter signal intensity and cognitive function in older adult hypertensive patients, white matter intensity was also positively correlated with cognitive function (64).

### Genetic polymorphisms and variants

Studies on genetic polymorphisms and variants have helped improve our understanding of the relationship between hypertension and cognitive decline. High serum levels of the ACE, which regulates BP, may play an important role in the incidence of amnestic MCI (aMCI) (65, 66). Middle-aged and older adult patients with aMCI who carry the allele coding for the high-activity variant (D) of ACE show greater cognitive impairment, whereas those who carry the low-activity allele (I) have an increased risk of dementia (67–69). Furthermore, angiotensin II type 1 receptor polymorphisms play an important role in BP regulation and are related to reductions in prefrontal and hippocampal volumes, hippocampal volume over time, and memory loss in older adults (70, 71).

The methylenetetrahydrofolate reductase (MTHFR) C677T polymorphism has been reported to be associated with hypertension (72). Studies have shown that riboflavin, a cofactor of MTHFR, induces hypertension by regulating the methylation level of homozygous genes (73). The regulators of the G protein signaling 2 1891-1892del TC and cytochrome P450 family 4 subfamily A member 11 T8590C polymorphisms increased the risk of hypertension (74). Another study showed that hypertensive patients with cognitive impairment carry a copy of the apolipoprotein E4 (ApoE4) gene (75).

### High homocysteine-induced cognitive decline in patients with hypertension

High homocysteine level promotes the occurrence of essential hypertension (EH) and cognitive impairment (76–78). It has been shown that the incidence of cognitive dysfunction increases as the concentration of homocysteine (Hcy) increases (79). Increased Hcy is likely to lead to vascular dementia, AD, and other pathological changes (78–80). Moreover, HHcy-stimulated vascular smooth muscle cell proliferation has been found to cause endothelial cell injury (81). The increase in Hcy leads to the oxidation of the vascular endothelium, which reduces vascular elasticity and increases susceptibility to hypertension (73, 79).

Cerebrovascular injury and neurotoxicity accelerate cognitive impairment. Hcy neurotoxicity manifests via the promotion of neuronal apoptosis, which affects nerve conduction and impairs cognitive function (79, 80, 82, 83). The connection between HHcy and cognitive decline can be explained by several mechanisms. HHcy, as a neurotoxin, promotes neurodegeneration via apoptosis and DNA breakage (82). Indeed, studies have shown that HHcy contributes to cognitive decline (83, 84). Additionally, recent studies have demonstrated that hypertensive patients with HHcy exhibit white matter hyperintensity and gray matter loss due to damaged cerebral vessels, which reflect the neurotoxic effects of HHcy (85). In mice, the Hcy level has been shown to affect the excitability of neurons and impair cognitive function. Moreover, long-term exposure to Hcy can induce changes in spatial learning, hippocampal signaling, and synaptic plasticity (84). Folic acid, vitamin B6, and vitamin B12 supplementation for 14 weeks significantly reduced the total serum Hcy level and improved cognitive function in middle-aged and older adult patients with HHcy (79, 86).

## Mechanism of the mediation of cognitive decline induced by hypertension via high homocysteine-dependent DNA methylation

Homocysteine regulates the expression of genes related to cognitive function by interfering with methyl group metabolism and epigenetic regulation. HHcy mediates hypomethylation, which is caused by impaired DNA transmethylation, resulting in cognitive decline (82).

### Homocysteine is an intermediate in the DNA methylation pathway

**FIGURE 1:**
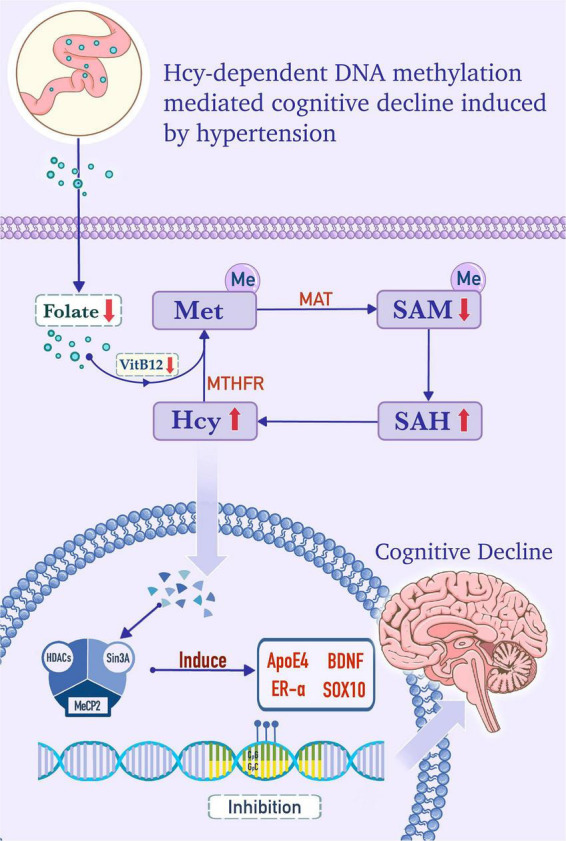
Homocysteine (Hcy)-dependent DNA methylation mediates cognitive decline induced by hypertension. In the cytoplasm, folate, from the serum as a substrate, and vitamin B12, as a cofactor, provide methyl for the conversion of homocysteine (Hcy) to methionine (Met). Met is converted into adenosylmethionine (SAM) by the catalysis of the Met adenosyltransferase (MAT). SAM is catalyzed into *S*-adenosylhomocysteine (SAH) by methyltransferase, and SAH is subsequently hydrolyzed into Hcy. Hcy is remethylated to form Met, and this step is dependent on methylenetetrahydrofolate reductase (MTHFR), folate, and vitamin B12. In hypertensive patients, folate and vitamin B12 deficiency, MTHFR deficiency, and/or MAT I/III deficiency, which are caused by MAT1A gene mutations, can increase the level of Hcy and promote the formation of the DNA methylation complex. The methylation complex is formed by methyl-CpG binding protein 2 (MeCP2), which recruits histone deacetylase (HDACs) and Sin3 transcription regulator family member A (Sin3A) under the catalysis of DNA methyltransferase (DNMT). The complex induces hypermethylation of apolipoprotein E4 (ApoE4), brain-derived neurotrophic factor (BDNF), estrogen receptor alpha (ER-α), and SOX10 genes, which is in accord with the lower expression levels of these factors and cognitive decline.

Methionine (Met) is a methyl donor that participates in the methylation of substrates, such as phospholipids, myelin, nucleic acids, choline, and catecholamine (79). Hcy is an intermediate product of the Met metabolism pathway. Met is converted into adenosylmethionine (SAM), which is catalyzed into *S*-adenosylhomocysteine (SAH) by methyltransferase. Subsequently, SAH is hydrolyzed to Hcy. Hcy can be remethylated into Met, and this reaction is dependent on folate as a substrate and vitamin B12 as a cofactor, as shown in Figure 1. Serum folate, Vitamin B12, and Hcy levels have been shown to correlate with cognitive function (79, 86). Moreover, HHcy is positively correlated with the prevalence of cognitive impairment and is considered a predictor of cognitive change (87, 88).

Homocysteine remethylation disorders are rare inherited disorders caused by deficient activity of the enzymes involved in the remethylation of Hcy to Met (89). Polymorphisms of the MTHFR gene are strongly associated with hypertension, and Met synthase 2756A > G and 5-methyltetrahydrofolate-homocysteine methyltransferase reductase 66A > G polymorphisms related to folate metabolism may serve as genetic markers for hypertension risk (90, 91). Patients with MTHFR deficiency exhibit cognitive deficits and psychiatric symptoms. In one study, the methylation rate of the methylenetetrahydrofolate dehydrogenase 1 (MTHFD1) promoter in patients with hypertension was shown to be significantly higher than that in the control group (92).

Homocysteine is a neurotoxic amino acid that induces calcium influx. Hcy-induced *N*-methyl-D-aspartic acid receptor channel activation can eventually lead to neuronal degeneration via glutamate excitotoxicity (93). HHcy reduces the cellular level of SAM, and co-treatment with SAM can antagonize apoptosis, which suggests that methylation mediates apoptosis (94). HHcy damages cerebral blood vessels via its neurotoxic effects and promotes the development of cognitive impairment and dementia. Some drug interventions for HHcy may improve and delay the progression of cognitive dysfunction and dementia. Patients with a folic acid deficiency with a normal Met level show impairments in spatial memory and learning. However, such deficits can be prevented by supplementation with Met in individuals with a folate-deficient diet (95). Furthermore, an increased serum Hcy level proportionally increases the risk of cognitive decline, whereas high levels of folic acid and B12 inhibit Hcy and serve as a protective factor for cognitive impairment. These findings suggest that increasing daily folic acid and vitamin B12 intake may normalize the Met cycle and methylation and protect the brain from functional damage (96). However, several studies have shown that vitamin supplements do not provide any benefit to patients with cognitive impairment (97). Thus, further investigations are necessary before interventions to reduce plasma Hcy levels are used to improve cognitive function.

### Aberrant DNA methylation in neurons is involved in hypertension-related cognitive decline

The estrogen receptor alpha (ER-α) gene promoter is hypermethylated in EH patients and is positively correlated with plasma Hcy level (98). Increased plasma Hcy levels in EH patients occur via the hypermethylation of the ER-α gene promoter region. A plausible mechanism for this process is that high levels of plasma Hcy in EH patients increases the metabolite SAM, which provides more methyl groups for DNA methylation and increases the activity of DNA methyltransferase (DNMT). This leads to hypermethylation of the ER-α promoter region (98). In atheromatosis patients, the ER-α gene promoter is hypermethylated and is positively correlated to plasma Hcy levels (99). Moreover, the ER-α-A gene promoter has a high methylation rate in white matter-hyperintense patients with cognitive impairment and is associated with plasma Hcy levels. Methylation of the ER-α-A gene is a significant determining factor for cognitive impairment and is also significantly correlated with serum Hcy levels and white matter hyperintensity. In addition, ER-α-A gene promoter methylation is higher in patients with cognitive dysfunction and is also related to high plasma Hcy levels (100).

When the RAAS system is activated, angiotensin II plays an important role in cognitive decline resulting from chronic intermittent hypoxia-induced hypertension (101). Promoter methylation inhibits the expression of the angiotensin II type 2 receptor (AT2R) gene, which is related to increased cerebral hypoxic-ischemic injury caused by perinatal stress in neonatal rats (102). It has been shown that hypermethylation of CpG islands in the promoter region of the androgen receptor gene is significantly correlated with the level of HHcy in patients with vascular cognitive impairment (103).

The hippocampus plays a crucial role in spatial learning and memory. Hypertension impairs adult hippocampal neurogenesis, CA1 neuron dendritic arborization, and long-term memory. Aldosterone levels are increased in patients with hypertension, and it has been shown that aldosterone treatment significantly decreases DNA methylation and BDNF expression in the hippocampus (104). Demethylation of the BDNF exon IV promoter causes phosphorylation of methyl-CpG binding protein 2 (MeCP2), which can activate the transcription of BDNF in the rat hippocampus (105). Furthermore, elevated methylation of the BDNF promoter predicts conversion from aMCI to AD (106). However, the methylation rate of the BDNF promoter has been reported to vary among Xinjiang Uygur and Han populations with MCI (107).

The role of neurotrophic factors in the regulation of hippocampal long-term potentiation (LTP), which is the sustained increase in excitatory synaptic strength that is fundamental to learning and memory, has received considerable attention. Hypobaric hypoxia exposure increases the expression of DNMT1 and DNMT3b and significantly decreases the levels of pMeCP2 and BDNF (108). DNMT1 and DNMT3a affect learning and memory by regulating DNA methylation and neuronal gene expression (109).

Simulation experiments in zebrafish have shown that long-term exposure to Met significantly increases the activity of acetylcholinesterase, which is an enzyme that indirectly negatively impacts cognitive performance and contributes to the occurrence of neurodegenerative diseases (110). When Met is administrated to mice, the levels of Hcy and the induction of DNA hypermethylation increase, which leads to the down-regulation of several gamma-aminobutyric acidergic neuronal markers, such as reelin and glutamic acid decarboxylase 67 (GAD67); moreover, correlates with the onset of cognitive decline and schizophrenia-like behaviors (111). Met-induced hypermethylation of the reelin and GAD67 gene promoters is effectively reversed by valproate, histone deacetylase inhibitors, clozapine, and sulpiride (111–113). In addition, during fear conditioning, the synaptic plasticity gene reelin is demethylated and activated, and the memory suppressor gene protein phosphatase 1 is hypermethylated and inhibited (114). Studies have also shown that DNA activity-dependent methylation and demethylation are important substrates for reward-related experience-driven behavior and neuronal plasticity (115).

### Homocysteine-dependent DNA methylation in oligodendroglial and/or myelin deficits

Genetic defects in enzymes involved in Hcy metabolism and folic acid, vitamin B6, or B12 deficiency elevate plasma Hcy levels, which increases the incidence of cardiovascular diseases (77, 79). The Met adenosyltransferase (MAT) 1a (MAT1A) gene, which encodes the major hepatic forms of the MAT protein, MATI and III, has been found to be strongly associated with hypertension (116). Patients with hypermethioninemia, which is caused by a MAT1A mutation, show neurological dysfunctions, cognitive impairment, learning impairment, and memory loss (117). MATI/III deficiency in mice due to a MAT1A gene mutation is characterized by high plasma Hcy levels and demyelination in the nervous system (118). The products of MAT and SAM are major methyl donors for myelin phospholipids, phosphatidylcholine, and sphingomyelin. Brain MRI studies have revealed periventricular hyperintensity and delayed or impaired myelination. Moreover, sural nerve biopsy studies have shown myelinated fiber loss (116–118).

Met is involved in the formation of the myelin sheath, which plays an important role in cognitive function. We found that Met-induced global DNA hypermethylation leads to oligodendroglial and/or myelin deficits and hypermethylation of the sex-determining region of the Y-chromosome (SRY)-related HMG-box 10 (SOX10) gene, which is an important factor for terminal differentiation of oligodendrocytes and cognitive behavioral changes (119). In addition, studies have demonstrated that Met can induce hypertension in mice (91, 120, 121). These findings suggest that DNA methylation may enable the regulation of cognitive dysfunction in patients with hypertension, although additional studies are needed to confirm this.

Hypertension induces cognitive decline and cerebral small vessel diseases, such as white matter hyperintensities, lacunar infarcts, and microhemorrhages. In white matter, DNA methylation deregulates ApoE4, huntingtin interacting protein 1, lectin mannose-binging 2, and myelin-associated oligodendrocytic basic protein (122, 123). DNA methylation of the SOX10 gene affects the gene expression of oligodendrocytes, which has become an epigenetic marker for schizophrenia (124).

The ApoE4 gene is associated with both hypertension and cognitive impairment induced by WMLs (75). MCI is part of the progression from normal aging to AD, and plasma Hcy and the ApoE4 gene are sensitive biomarkers for MCI in AD patients. Reduced ApoE methylation increases plasma ApoE levels, and increases in CPG165, CPG190, and CPG198 methylation are risk factors for MCI. Furthermore, increased CPG227 methylation decreases plasma ApoE levels, which results in a decreased risk of MCI (125).

## Conclusion and future perspectives

Previous studies have established that hypertension induces cognitive decline via cerebral blood flow perfusion imbalance, white and gray matter injury with BBB disruption, neuroinflammation, and Aβ deposition. In addition, HHcy is an independent risk factor for hypertension-induced early cognitive impairment (126). However, the exact molecular mechanism underlying the effect of HHcy on cognitive impairment induced by hypertension remains unknown.

This review described changes in Hcy-dependent DNA methylation products, such as MAT, SAM, SAH, and MTHFR. Aldosterone treatment significantly decreases global DNA methylation, ER-α and ER-α-A gene promoter hypermethylation in EH patients, and white matter hyperintensity in patients with cognitive impairment, Furthermore, BDNF and ApoE methylation are involved in the MCI and AT2R receptor gene hypermethylation in the developing brain (98, 109). Met induces hypertension in mice, and we previously showed that DNA hypermethylation leads to cognitive behavioral changes with oligodendroglial and/or myelin deficits in Met-induced mice. These findings are shown in Tables 1, 2.

**TABLE 1:** Genes related to cognitive decline induced by hypertension.

Genes	Cognitive function	Exposure	Correlation with hypertension	References
Hcy	Cognitive impairment	Hypertension	Hcy promotes the occurrence of essential hypertension	(84–86, 88, 130)
AT2	Cognitive decline	Chronic intermittent hypoxia-induced hypertension	Activation of RAAS contributed to hypertension	(101)
AR	Cognitive impairment	Vascular cognitive impairment (VCI)	Androgens induce hypertension, the polymorphic CAG repeat in androgen receptor is associated with hypertension	(103)
Genes in the hippocampus	Spatial learning and memory	Aldosterone	Activation of RAAS system contributed to the development of hypertension	(104)
ACE	ACE inhibitors suppress cognitive function	Hypertension	ACE influences the metabolism of angiotensin in the RAAS system	(28)
ER-α-A gene	Cognitive impairment	White matter hyperintensity	Correlated with plasma HHcy level	(100)
ApoE4	Cognitive impairment	Hypertension	Hypertension is associated with cognitive deficits in individuals who possess a copy of ApoE4 gene	(75)

RAAS, renin-angiotensin-aldosterone system; ACE, angiotensin-converting enzyme; AR, androgen receptor; AT2, angiotensin II; ApoE4, apolipoprotein E4.

**TABLE 2:** DNA methylation in cognitive decline and/or hypertension related diseases.

Genes with greatest association		Exposure	Methylation status	Types	References
Inter-mediate product of met conversion to Hcy	Hcy	Hypertension	Hcy increase	Human	(84–86, 88, 130)
	SAM SAH	Met	SAM increase SAH decrease	Folate-deficient rats	(95)
	5mC	Met	5mC increase	Mice	(119)
	MAT I/III	Hypermethioninemia	Methionine adenosyltransferase deficiency	MAT I/III deficiency mice	(118)
DNMT1		Fear conditioning	DNA methylation maintenance	Mice	(109)
DNMT3A		Dnmt3a–/– NSCs	Methylation changes of MBP, PLP1, Olig1, Id2/4	Mice	(131)
AR		Vascular cognitive impairment (VCI)	Methylation status of CpG islands in the promoter region of AR gene	Human	(103)
AT2R		Perinatal stress and hypoxic ischemic encephalopathy	CpG methylation at AT2R gene promoter	Neonate rats	(102)
Genes in the hippocampus		Aldosterone	Aldosterone treatment significantly decreased global DNA methylation in the hippocampus	Mice	(104)
ACE		Hypertension	The hypermethylation of ACE II gene was associated with hypertension	Human	(132, 133)
ER-α		Hypertension	Hypermethylation	Human	(98)
		Atheromatosis (AS)	The CpG island of ER-α gene promoter region was highly methylated, correlated with blood Hcy concentration in AS patients	Human	(134)
ER-α-A gene		White matter hyperintensity (WMH)	Methylation status of CpG islands in ERsmall a, Cyrillic-A gene promoter was analyzed by nested methylation-specific PCR	Human	(100)
BDNF		Valproic acid	BDNF DNA demethylation in the hippocampus of valproic acid-treated group	Rat	(104)
		Amnestic mild cognitive impairment (aMCI)	DNA methylation levels of in promoter I, promoter IV of BDNF gene were significantly higher in the aMCI group	Human	(106)
		Exercise	Demethylation of the BDNF exon IV promoter caused an increase in BDNF mRNA and protein in the hippocampus	Rat	(105)
		Epilepsy	Met increases DNA methylation in promoter IV and exon IV of BDNF gene	Rat	(135)
ApoE4		Mild cognitive impairment (MCI)	Methylation of CpGs 165, 190, and 198 were high risk factors, higher CpG-227 methylation correlated with a lower risk for MCI	Human	(125)
HIP1, LMAN2, MOBP		Multiple system atrophy (MSA)	Illumina methylation EPIC arrays results showed DNA methylation changes with 157 CpG sites and 79 genomic regions. HIP1, LMAN2 and MOBP were amongst the most differentially methylated loci.	Mice	(123)

Met, methionine; SAM, adenosylmethionine; SAH, *S*-adenosylhomocysteine; MTHFD1, methylenetetrahydrofolate dehydrogenase 1; ACE-Is, angiotensin-converting enzyme inhibitors; AR, androgen receptor.

Widespread aberrant DNA methylation is present in patients with hypertension, and the level of 5-methylcytosine (5mC) and DNMTs, methylation of the MTHFD1 promoter, RAAS such as the ACE, angiotensin type 1 receptors AT1a and AT1b, endothelin-converting enzyme-1, adducin 1, the renal sodium retention system(e.g., 11 beta-hydroxysteroid dehydrogenase 2, and sodium-potassium-chloride cotransporter 1), the sympathetic nervous system(e.g., norepinephrine transporter), fatty acid binding protein 3, and glucokinase gene methylation are affected (92, 127). Moreover, DNA methylation mediates the process of cognitive decline. Furthermore, changes in DNMT1, DNMT3a2, DNMT3b, MeCP2, and 5mC correlate with cognitive decline (128).

Methylation regulates the expression of genes involved in the pathogenesis of hypertension (92, 127), some of which also mediate cognitive impairment (108, 128, 129). However, few studies have directly demonstrated that methylation regulates cognitive dysfunctions in patients with hypertension, such as learning, memory formation, and behavioral plasticity deficits. Methylation of numerous related genes, such as ACE, AT1a and AT1b in the RAAS, and SOX10, has positive effects on cognitive decline and provides a focus for future research on cognitive decline in patients with hypertension caused by DNA methylation. Furthermore, products of the Met metabolism pathway, such as SAM, SAH, Met, Hcy, folate, and vitamin B12, may be studied further in regard to cognitive decline induced by hypertension.

## Author contributions

CW and R-YZ searched the literature and drafted the manuscript. X-SC critically revised the manuscript. All authors contributed to the article and approved the submitted version.

## Abbreviations

ACE: angiotensin-converting enzyme
AD: Alzheimer’s disease
ApoE4: apolipoprotein E4
AT2R: angiotensin II type 2 receptor
Aβ: amyloid beta
BBB: blood–brain barrier
BDNF: brain-derived neurotrophic factor
BP: blood pressure
Dnmts: DNA methyltransferase
EH: essential hypertension
ER-α: estrogen receptor alpha
Hcy: homocysteine
HHcy: high homocysteine
MAT1A: methionine adenosyltransferase 1a
5mC: 5-methylcytosine
MCI: mild cognitive impairment
MeCP2: methyl-CpG binding protein 2
Met: methionine
MRI: magnetic resonance imaging
MTHFD1: methylenetetrahydrofolate dehydrogenase 1
MTHFR: methylenetetrahydrofolate reductase
PET: positron emission tomography
RAAS: renin-angiotensin-aldosterone system
RAGE: receptor for advanced glycation end products
SAM: *S*-adenosyl methionine
SAH: *S*-adenosylhomocysteine
SOX10: sex determining region of the Y-chromosome (SRY)-related HMG-box 10
VCI: vascular cognitive impairment
WMLs: white matter lesions.
